# Impact of different financial incentive structures on a web-based health survey: do timing and amount matter?

**DOI:** 10.20517/chatmed.2023.002

**Published:** 2023-07-03

**Authors:** Jaclyn N. Escudero, Jasmin A. Tiro, Diana S.M. Buist, Hongyuan Gao, Tara Beatty, John Lin, Diana L. Miglioretti, Rachel L. Winer

**Affiliations:** 1 Department of Global Health, University of Washington, Seattle, WA 98195, USA.; 2 Department of Public Health Sciences, University of Chicago, Chicago, IL 60637, USA.; 3 Kaiser Permanente Washington Health Research Institute, Seattle, WA 98101, USA.; 4 Department of Epidemiology, University of Washington, Seattle, WA 98195, USA.; 5 Division of Biostatistics, Department of Public Health Sciences, University of California Davis School of Medicine, Davis, CA 95616, USA.

**Keywords:** Conditional, costing, incentive, survey, unconditional

## Abstract

**Aim:**

Financial incentives improve response to electronic health surveys, yet little is known about how unconditional incentives (guaranteed regardless of survey completion), conditional incentives, and various combinations of incentives influence response rates. We compared electronic health survey completion with two different financial incentive structures.

**Methods:**

We invited women aged 30-64 years enrolled in a U.S. healthcare system and overdue for Pap screening to complete a web-based survey after receiving a mailed human papillomavirus (HPV) self-sampling kit in a pragmatic trial. HPV kit returners (*n* = 272) and non-returners (*n* = 1,083) were allocated to one of two different incentive structures: (1) Unconditional: $5 pre-incentive only (*n* = 653); (2) Combined: $2 pre-incentive plus $10 post-incentive conditional on completion (*n* = 702). Chi-square tests evaluated whether survey completion differed by incentive structure within kit return groups or was modified by kit return status. For each incentive-by-kit status group, the cost-per-survey response was calculated as: ([number invited*pre-incentive amount] + [number responses*post-incentive amount]) / number responses.

**Results:**

Overall, survey response was higher in kit returners vs. kit non-returners (42.6% *vs.* 11.0%, *P* < 0.01), and survey response was higher in the combined (20.1%) *vs.* unconditional (14.4%) incentive group (*P* = 0.01). Kit return status did not modify the association between incentive type and survey response (*P* = 0.52). Among respondents, time to survey completion did not differ by incentive type among either kit returners or non-returners. Among returners, the cost-per-survey response was similar between groups ($13.57 unconditional; $14.15 combined); among non-returners, the cost was greater in the unconditional ($57.78) versus the combined ($25.22) group.

**Conclusion:**

A combined incentive can be cost-effective for increasing survey response in health services research, particularly in hard-to-reach populations.

## INTRODUCTION

Surveys on subgroups in larger trials can provide critical information to help interpret findings and support translation of interventions into practice^[1]^. Surveys are also the most efficient quantitative research method to collect information from large numbers of individuals in population-based settings^[2]^. However, low response rates can bias results and decrease generalizability^[3]^. Response rates are correlated with demographic factors, including participant age, sex, and socioeconomic status^[4]^. Additionally, response rates are influenced by survey design features, including length and format, recruitment and invitation methods, content and style of questions, and type and timing of incentives offered for participation^[5,6]^. While financial incentives increase survey response^[7,8]^, less is known about the relative influence of unconditional incentives (guaranteed regardless of survey completion), conditional incentives (guaranteed after survey completion), and other various combinations. Unconditional incentives are generally more effective than conditional incentives for increasing survey response^[5,9-11]^. To our knowledge, however, only one health-related study surveying healthcare consumers has evaluated the influence of a combined incentive structure in which individuals are offered both an unconditional pre- and conditional post-incentive^[12]^, and none have specifically compared a combined pre/post-incentive with an unconditional pre-incentive alone. Combined incentives incur lower upfront implementation costs when small denomination pre-incentives are used; thus, it is important to determine if these incentive types yield similar or higher response rates compared to larger-denomination unconditional incentives. Edwards *et al.*’s 2009 meta-analysis found no response differences between unconditional and conditional incentives for electronic questionnaires, or when larger versus smaller financial incentives were used^[5]^. Other studies have evaluated conditional and unconditional incentives for postal and telephone-based surveys. A 2018 systematic review focused on studies involving health-related questionnaires concluded that unconditional monetary incentives were more effective than conditional incentives in increasing response rates in postal surveys, among both patients (response ratio: 1.15; 95%CI: 1.09, 1.21) and nonpatients (response ratio: 1.24; 95%CI: 1.12, 1.38)^[9]^. In a telephone-based survey of postpartum women, Beydoun *et al.* found a combined $5 telephone card pre-incentive and $25 post-incentive was more effective than a $30 conditional incentive for increasing telephone tracing rates, with no difference in survey completion^[12]^.

Studies have demonstrated mixed results related to an incentive’s effect on response time. Parkes *et al.* showed that unconditional incentives significantly reduced survey response times compared to those who received no incentive^[13]^. Blomberg *et al.* compared conditional and unconditional lottery tickets and found that participants who received a lottery ticket unconditionally took more time to respond and were least likely to respond^[14]^.

We nested a comparison of two incentive structures in a randomized trial evaluating a home-based human papillomavirus (HPV) testing strategy to increase cervical cancer screening in underscreened women (a hard-to-reach population)^[15,16]^. We invited two subgroups of women (those who did and did not return HPV kits) to complete a survey on their perspectives on this screening modality and allocated them to either an unconditional or combined incentive. We compared the effect of a $5 unconditional incentive (pre-incentive) with a combined $2 unconditional incentive mailed with the invitation letter plus a $10 conditional incentive sent after completing the survey (hereafter called combined incentive) on survey response, as well as the cost implications of these incentive strategies.

## METHODS

### Study Design

From January to July 2015, we conducted a web-based survey with a subset of 30-64-year-old women who were mailed an HPV self-sampling kit six months earlier as part of the Home-based Options to Make cervical cancer screening Easy (HOME) pragmatic trial at Kaiser Permanente Washington (KPWA) (ClinicalTrials.gov identifier: NCT02005510)^[15]^. Invitations were mailed weekly to women who did (*n* = 272) and did not (*n* = 1,083) return a kit until we reached the target sample size of 100 per group. All eligible participants were allocated to receive either an unconditional $5 pre-incentive only (*n* = 653) or combined incentive [unconditional $2 bill plus $10 conditional incentive upon survey completion] (*n* = 702). These amounts were determined based on feasibility and ensuring the pre-incentive was not coercive.

Invitations included a research information sheet, instructions with a URL to access the survey and a QR code to scan, and a $2 or $5 cash pre-incentive with an explanation of the conditional incentive ($10) if allocated to the combined group. The research information sheet described a 5-10-minute web survey on experiences with a “health screening kit” mailed 6 months prior with a toll-free telephone number to call with questions, request a paper version of the survey, or “opt-out” of having their individual-level medical record data used for research. Initially, women who did not respond within 1-2 weeks received up to three telephone reminder calls and one voicemail over a 10-day period asking if the invitation was received and an offer to mail a paper version or e-mail the survey link. Invitees who did not complete a survey were automatically mailed a paper version after six weeks. After observing low survey response rates following telephone reminders and automatic paper survey mailings, these strategies were discontinued after two and three months, respectively. The protocol was approved by the KPWA Institutional Review Board.

### Data Analysis

We used chi-square tests to compare the distribution of select covariates from electronic medical record (EMR) data (age, race, ethnicity, census block household income, and Charlson comorbidity score^[17]^) by incentive structure randomization group, separately for women who returned and did not return a kit. A chi-square test was also used to assess whether survey completion (yes/no) varied by incentive type (unconditional versus combined), and a chi-square test of homogeneity was used to evaluate whether the association between survey completion and incentive type was modified by kit return status. Among women who returned the survey, we used a Wilcoxon Rank-Sum test to examine whether the number of days between invitation mailing and survey completion varied by incentive type. All statistical tests were two-sided with alpha of 0.05. Analyses were conducted using SAS version 9.4.

For each incentive-by-kit status group, we calculated cost-per-survey response as: ([number invited*pre-incentive amount] + [number responses*post-incentive amount]) / number responses.

## RESULTS

### Survey participation

Most women invited to the survey were aged 50-64 years, non-Hispanic, white, and had a Charlson comorbidity score of 0. Within kit-returners and non-returners, distributions of EMR-derived covariates were similar between women randomized to the unconditional versus combined incentive [Table 1]. Of the 235 completed surveys, 192 were web-based and 43 were on paper. Overall, survey response was higher in kit returners *vs.* non-returners [(116/272) 42.6% *vs.* (119/1,083) 11.0%, *P* < 0.001], and higher in the combined ([141/702] 20.1%) *vs.* unconditional [(94/653) 14.4%] group (*P* = 0.01) [Table 2]. Kit return status did not significantly modify the association between incentive type and survey response (*P* = 0.52). Survey response was not statistically significantly higher in the combined versus unconditional group among kit-returners [(67/139) 48.2% *vs.* (49/133) 36.8%, *P* = 0.06] and higher among non-returners [(74/563) 13.1% *vs.* (45/520) 8.7%, *P* = 0.02]. Among respondents, time to survey completion did not differ by incentive type among either kit returners [combined: median [interquartile range (IQR)] = 8 (4-22) days *vs.* unconditional: median (IQR) = 9 (5-22) days; *P* = 0.64] or non-returners [combined: median (IQR) = 14.5 (6-44) days *vs.* unconditional: median (IQR) = 14 (5-26) days; *P* = 0.35].

### Survey costs

Overall, the incentive cost-per-survey response was lower in the combined $2/$10 ($19.96) versus the unconditional $5 ($34.73) incentive group [Table 3]. Among kit returners, however, cost-per-survey response was similar between groups: $14.15 combined versus $13.57 unconditional. Among kit non-returners, the cost-per-survey response was less than half in the combined ($25.22) versus the unconditional ($57.78) group.

## DISCUSSION

Survey response rates were higher in women allocated to a combined incentive versus an unconditional pre-incentive only. Our survey was embedded in a randomized pragmatic trial that was designed to evaluate an intervention to increase cervical cancer screening uptake among members of an integrated healthcare delivery system who were underscreened and, therefore, relatively less engaged in the healthcare system than screening-adherent women^[15]^. Though response rates were much lower in kit non-returners, the combined incentive was associated with higher response in both groups and less costly than the unconditional only incentive among kit non-returners, the hardest-to-reach subgroup.

We found a combined $2/$10 incentive (i.e., small pre-incentive amount) resulted in higher response rates and lower total costs than a $5 unconditional incentive, when response rates were relatively low. While we did not find any web-based surveys comparing unconditional and combined incentives, several studies have evaluated the relative influence of unconditional versus conditional incentives on survey response^[5,9]^. Though some studies have suggested a possible disparity between paper and web-based survey response rates, our study did not compare the two formats^[9]^.

Receiving an unconditional financial incentive in advance of participation may foster trust and goodwill, thereby motivating a subject to return the favor by completing the survey^[18]^. In our study, all subjects received an unconditional incentive. The $2 bill, a less common denomination that grabs potential participants’ attention^[11,19]^, and the added post-incentive may have further encouraged members of the combined incentive group to complete our survey. Edwards *et al.*’s 2009 meta-analysis found no response differences between unconditional and conditional incentives for electronic questionnaires, or when larger versus smaller financial incentives were used^[5]^. We are unaware of any other studies that have specifically compared the influence of unconditional pre-incentives with combinations of pre- and post-incentives in an electronic survey of healthcare consumers, and how these structures influence survey costs.

A short time to survey return may be important, especially if evaluation of an intervention is time sensitive. In our study, incentive type did not influence survey response time. Other studies suggest that response time may be dependent on incentive structures, types of incentives, or what the research participation entails^[13,14]^.

There is value in including methodological studies to improve the design and yield of sub-studies conducted within pragmatic trials. While our sub-study was not originally designed to examine different incentive structures, we leveraged an opportunity to embed a methodological study within the survey sampling design. Other studies evaluated multiple incentive strategies^[5,9]^, and randomized more than 1,000 participants per group. In comparison, our sample was relatively small and precluded evaluating greater than 2 incentive structures. We encourage others to consider evaluating other pre/post-incentive combinations.

Reflective of the underlying parent trial population, survey invitees were mostly white and non-Hispanic, and all were insured members of an integrated healthcare system. Thus, our findings may not be generalizable to other populations (e.g., underrepresented racial/ethnic groups, uninsured people). The incentive structures used in this study may also perform differently among populations with other health conditions or in research with non-survey-based data collection.

## CONCLUSION

Our results suggest that combination incentives are preferable to unconditional only incentives for increasing response to health-related surveys. Furthermore, when low response rates are expected, offering a small pre-incentive combined with a larger post-incentive could be more cost-effective than offering only a larger pre-incentive. Future health services research seeking to survey hard-to-reach populations may want to consider using combined incentives. It would also be worthwhile to explore the use of combined incentives to increase engagement in other types of health studies and interventions, such as digital health.

## Figures and Tables

**Table 1.** Baseline characteristics by kit return status and incentive structure

	Kit returners (N = 271^a^)					Kit non-returners (N = 1079^b^)				
	$5 (N = 132)		$2/10 (N = 139)			$5 (N = 519)		$2/10 (N = 560)		
Covariates^c^	n	(%)	n	(%)	P-value^d^	n	(%)	n	(%)	P-value^d^
**Age Group, years**										
30-39	14	(10.6)	20	(14.4)	0.30^e^	88	(17.0)	97	(17.3)	0.97^e^
40-49	33	(25.0)	25	(18.0)		137	(26.4)	150	(26.8)	
50-64	85	(64.4)	94	(67.6)		294	(56.6)	313	(55.9)	
**Race**										
White	102	(77.3)	111	(79.9)	0.19^f^	380	(73.2)	395	(70.5)	0.41^e^
Asian	7	(5.3)	13	(9.4)		40	(7.7)	59	(10.5)	
Black/African-American	8	(6.1)	3	(2.2)		20	(3.9)	15	(2.7)	
Other^g^	9	(6.8)	10	(7.2)		42	(8.1)	50	(8.9)	
Unknown	6	(4.5)	2	(1.4)		37	(7.1)	41	(7.3)	
**Ethnicity**										
Non-Hispanic	121	(91.7)	132	(95.0)	0.50^f^	465	(89.6)	489	(87.3)	0.26^e^
Hispanic	5	(3.8)	4	(2.9)		18	(3.5)	31	(5.5)	
Unknown	6	(4.5)	3	(2.2)		36	(6.9)	40	(7.1)	
**Census Block Household Annual Income**										
$0-49,999	31	(23.5)	28	(20.2)	0.66^e^	120	(23.2)	117	(20.9)	0.70^e^
$50,000-74,999	47	(35.6)	43	(30.9)		188	(36.2)	187	(33.4)	
$75,000-99,999	33	(25.0)	41	(29.5)		127	(24.5)	143	(25.5)	
$100,000+	14	(10.6)	18	(12.9)		48	(9.2)	58	(10.4)	
Unknown	7	(5.3)	9	(6.5)		36	(6.9)	55	(9.8)	
**Charlson Comorbidity Index** ^h^										
0	101	(76.5)	110	(79.1)	0.13^f^	422	(81.3)	449	(80.2)	0.92^f^
1	21	(15.9)	14	(10.1)		56	(10.8)	62	(11.1)	
2	3	(2.3)	10	(7.2)		27	(5.2)	30	(5.4)	
3+	7	(5.3)	5	(3.6)		14	(2.7)	19	(3.4)	

a One kit returner opted out of medical record review after receiving a survey invitation and therefore baseline characteristics were not extracted; the participant is excluded from this table.

b Four kit non-returners opted out of medical record review after receiving a survey invitation and therefore baseline characteristics were not extracted; the participants are excluded from this table.

c Covariates were derived from electronic medical records and measured as of trial randomization date.

d Two-sided

e Chi-Square Test

f Fisher’s Exact Test (cell size <5)

g Includes American Indian/Alaska Native/Native Hawaiian, More than one race, and Other race categories

h Calculated from a weighted index of 19 comorbid conditions [7]

**Table 2.** Survey response by incentive structure and kit return status

	Overall			Unconditional Incentive Group ($5)			Combined Incentive Group ($2 unconditional plus $10 conditional)				
	Invited	Completed Survey		Invited	Completed Survey		Invited	Completed Survey		P-value^a^ (combined vs. unconditional)	P-value^b^ for interaction by kit return status
	N	n	(%)	N	n	(%)	N	n	(%)		
**All women**	1355	235	(17.3)	653	94	(14.4)	702	141	(20.1)	0.006	
Kit returners	272	116	(42.6)	133	49	(36.8)	139	67	(48.2)	0.058	0.515
**Kit non-returners**	1083	119	(11.0)	520	45	(8.7)	563	74	(13.1)	0.018	

a Chi-Square Test; two-sided

b Chi-Square Test of Homogeneity; two-sided

**Table 3.** Cost-per-survey response by incentive structure and kit return status

	Unconditional Incentive Group ($5)			Combined Incentive Group ($2 unconditional plus $10 conditional)		
	Completed Survey %	Cost-per-survey Response	Total Cost	Completed Survey %	Cost-per-survey Response	Total Cost
**All women**	14.4%	$34.73	$3,265	20.1%	$19.96	$2,814
**Kit returners**	36.8%	$13.57	$665	48.2%	$14.15	$948
**Kit non-returners**	8.7%	$57.78	$2,600	13.1%	$25.22	$1,866
